# Effect of Hyaluronic Acid on the Acceleration of Bone Fracture Healing: A Systematic Review

**DOI:** 10.3390/biomedicines13061353

**Published:** 2025-05-31

**Authors:** Helena Fuguet Surroca, Esther Caballé Pardo, Leonor Ramírez-Andrés, Elena Nieto-Gonzalez, Javier Ferrer-Torregrosa, Eduardo Nieto-Garcia

**Affiliations:** Podiatry Department, Faculty of Medicine and Health Sciences, Valencia Catholic University San Vicente Mártir, 46001 Valencia, Spain; helena.fuster@mail.ucv.es (H.F.S.); esther.caballe@mail.ucv.es (E.C.P.); leonor.ramirez@ucv.es (L.R.-A.); elena.nieto@ucv.es (E.N.-G.); eduardo.nieto@ucv.es (E.N.-G.)

**Keywords:** hyaluronic acid, bone consolidation, bone regeneration, fracture

## Abstract

**Background/Objectives:** Hyaluronic acid (HA) is a natural substance in the human body with anti-inflammatory and healing properties that help repair bone by supporting cell growth, blood vessel formation, and tissue structure. A common complication after minimally invasive surgery is delayed bone healing in osteotomies. HA may offer a useful treatment to support faster recovery. **Methods:** This systematic review followed PRISMA guidelines and was pre-registered in PROSPERO (ID: CRD420250654929). Searches were conducted in PubMed, EbscoHost, Web of Science, and Scopus up to 25 January 2025. Studies from the last five years on HA and bone healing were included. The main outcomes were faster bone repair and improved regeneration. Study quality was assessed using the OCEBM, ROBINS-I, and GRADE tools. **Results:** Out of 96 studies, 9 met the inclusion criteria. HA, especially when combined with other materials or stem cells, helped bone repair by supporting new bone formation. Materials like 3D-printed scaffolds, hydrogels, and meshes showed good results in bone healing. However, differences in the study design made direct comparison difficult. **Conclusions:** Hyaluronic acid shows promise for bone repair, especially in combination with other materials. More standardized clinical trials are needed to confirm its effectiveness and define how best to use it in minimally invasive surgeries.

## 1. Introduction

Minimally invasive foot surgery (MIS) has gained increasing relevance in recent years, with a growing number of procedures being performed due to its ability to achieve outcomes comparable to those of traditional open surgery, but with fewer postoperative complications and faster recovery times for patients [1]. MIS involves making small incisions to access and correct bone structures and soft tissues of the foot, thereby restoring its function and biomechanics. Among the procedures encompassed in MIS are osteotomies, either complete or incomplete, which, once performed, activate spontaneous bone healing mechanisms.

Approximately 2–10% of long bone fractures may present complications during the consolidation process, such as delayed union, malunion, or nonunion [2]. Although these complications are less frequent in minimally invasive foot surgeries, nonunion can still occur. Studies by Magnan et al., 2017 [3], McMurrich et al., 2020 [4], and Haque et al. [5]. have reported cases of nonunion in minimally invasive osteotomies, highlighting the relevance of exploring adjuvant therapies to enhance bone healing in this context.

These problems are often associated with factors such as insufficient blood supply, infection, instability at the fracture site, improper postoperative fixation, or the presence of systemic diseases [2,6,7,8]. Inadequate bone consolidation [9] leads to persistent pain and instability, negatively impacting the patient’s physical and mental well-being.

Several therapeutic alternatives have been proposed to improve bone consolidation, including the use of osteosynthesis materials, physical therapies such as magnetotherapy [10], low-level laser therapy [11,12], focused ultrasound [13], and the implementation of scaffolding techniques [14,15]. These techniques include both bone grafts and the application of specialized hydrogels [16,17], as well as technological innovations like 3D printing [18,19,20].

Among the historically predominant options, bone grafts, known as bone graft substitutes (BGSs) [20,21], have long been considered the gold standard. In recent years, 3D printing technology [22,23,24] has gained momentum in this field, particularly through the development of bioprinting techniques. This approach focuses on creating three-dimensional structures using bioinks composed of hydrogels, collagen, hyaluronic acid (HA) [21], and in some cases, cells and growth factors. These constructs aim to mimic the properties of natural bone by providing an extracellular-like environment, while also being biodegradable and resorbable. Despite advantages such as treatment customization and the ability to shape the material to specific needs, their limited mechanical strength remains a significant limitation, highlighting the need for further research to enhance their clinical functionality.

Therefore, the objective of this systematic review is to evaluate the effect of HA on the acceleration of bone fracture healing, with the aim of supporting its potential future application in surgically performed osteotomies to improve bone consolidation outcomes.

## 2. Materials and Methods

This systematic review was conducted in accordance with the criteria established by the PRISMA statement [25], ensuring adherence to methodological quality standards. The protocol was registered in PROSPERO (identification number CRD420250654929). The literature search was performed in the PubMed, EbscoHost, Web of Science, and Scopus databases on 25–27 February 2025, using terms related to “hyaluronic acid”, “bone regeneration”, and “osteotomy”. A summary of the systematic process is shown in Table 1.

### 2.1. Eligibility Criteria

#### 2.1.1. Inclusion Criteria

Studies conducted in humans within the past five years were included, with a focus on investigating the use of HA in bone consolidation. Interventions had to involve the application of HA, either alone or in combination with bone grafts or other biomolecules. Outcomes of interest included accelerated bone healing and enhanced bone regeneration, assessed through imaging techniques and histological analysis.

#### 2.1.2. Exclusion Criteria

Studies were excluded if they did not provide sufficient information on the application of HA, or if they were conducted exclusively on animal models without clinical correlation in humans. Systematic reviews and meta-analyses that did not contain primary data were also excluded, as they did not allow for direct evaluation of HA’s effects on bone consolidation. Additionally, publications in languages other than English or Spanish were excluded to ensure the accurate interpretation of the methodology and results. Lastly, studies in which HA was not the primary therapeutic agent under evaluation were not considered, ensuring the relevance and specificity of the included studies with respect to the objective of this review.

### 2.2. Search Strategy

The primary search focused on studies reporting the effect of HA on the acceleration of bone healing in fractures and minimally invasive foot osteotomies. The final search date was 25 February 2025. Searches were conducted in MEDLINE via PubMed, EbscoHost, Web of Science, and Scopus. The search strategy was constructed using the PICO framework and included terms related to HA intervention in bone fracture consolidation. The search string used in MEDLINE/PubMed is given below.

(“Hyaluronic Acid” OR Hyaluronan OR “Hyaluronate alginate” OR “Sodium Hyaluronate” OR “Hyaluronic Gel” OR Hyaluronate OR Hyaluronidase) AND (“bone regene*” OR “Fracture Healing” OR Osteogenesis OR “Bone Formation” OR “Bone Repair” OR “Bone Healing” OR “Bone Regeneration”) AND (Osteotom* OR “Orthopedic Procedures” OR “Percutaneous Procedures” OR Osteosynthes* OR “Bone Surgery” OR “Fracture Fixation” OR “Bone Fracture Treatment” OR Orthopedics).

Additional search strings for other databases are detailed in Appendix A and were adapted using the Polyglot Search Translator Tool.

### 2.3. Study Selection

To eliminate duplicate references, an online tool (https://www.sr-accelerator.com/#/deduplicator) accessed on 25–27 February 2025 was used first, followed by manual removal using the Mendeley reference manager. Two authors (H.F.S. and E.C.O.P.) independently screened the titles and abstracts to assess initial eligibility using an online platform (https://www.sr-accelerator.com/#/disputatron) on 25–27 February 2025. Any discrepancies were resolved through discussion, and if necessary, a third reviewer (J.F.T.) was consulted.

### 2.4. Study Coding and Data Extraction

Data extraction was carried out independently by two authors. From each included study, the following data were collected and coded: authors and year of publication, type of HA used, study design, interventions and control groups applied. In addition, results were recorded regarding bone regeneration, bone quality, and specific findings such as bone density and degree of angiogenesis. The extracted data were organized in an Excel spreadsheet to facilitate statistical and comparative analysis.

### 2.5. Methodological Quality and Risk of Bias

The methodological quality and risk of bias were independently assessed by two reviewers using the Grading of Recommendations Assessment, Development, and Evaluation (GRADE) system. GRADE is a widely recognized tool that provides a structured framework for evaluating the certainty of evidence and the strength of recommendations in biomedical research. This approach considers several factors, including study design, risk of bias, consistency of results, the applicability of evidence, and the precision of findings. The certainty of evidence is categorized into four levels (high, moderate, low, or very low), and recommendations can be either strong or conditional, depending on the balance of benefits and risks, evidence quality, and practical considerations. GRADE offers a rigorous and transparent framework to support evidence-based clinical decision making, ensuring that the conclusions of this review are interpreted with the highest possible reliability.

## 3. Results

### 3.1. Search Results

**Figure 1:**
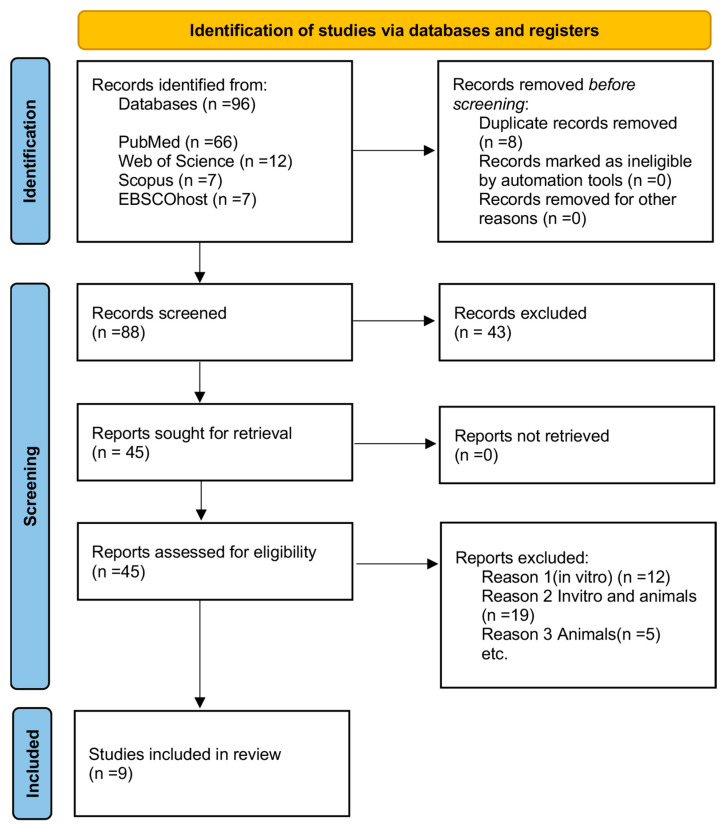
PRISMA 2020 flow diagram of evaluated studies.

Figure 1 illustrates the PRISMA flow diagram outlining the different stages of the search and study selection process for this review. The initial search in databases such as PubMed, EbscoHost, Web of Science (WOS), and Scopus yielded a total of 66 records from PubMed, 12 from WOS, 7 from Scopus, and 7 from EBSCO. Articles were subsequently excluded if the abstract did not indicate the use of HA for bone regeneration, or if HA was used for unrelated applications (e.g., cartilage or meniscus regeneration). Additionally, eight duplicate records were removed, as well as five systematic reviews that did not contain original research data. In total, 83 studies were excluded after abstract screening, resulting in 45 articles eligible for full-text assessment.

### 3.2. Main Findings

It is important to note that the included studies did not assess a standardized set of outcomes, which limits the direct comparability of results across studies and supports the use of a narrative synthesis approach in this review (see Table 2).

The reviewed studies highlight the use of HA as a key component in bone regeneration, particularly when combined with biomaterials or stem cells, enhancing its osteoconductive and osteoinductive properties. Both clinical evaluations in humans and preclinical studies in animal models were included, employing advanced techniques such as micro-computed tomography (micro-CT) and histomorphometric analysis to measure bone density, graft integration, and joint function. Methodological designs varied from case series [23,24] (e.g., Özdemir 2023; Çağlar Kir 2019) to randomized controlled trials (RCTs) [25,26] (Park 2016; Velasco-Ortega 2021). Park et al. (2016) demonstrated that the combination of mesenchymal stem cells (MSCs) and HA supports cartilage regeneration without adverse effects [25], while Na et al. (2020) found that Cartistem (umbilical cord-derived MSCs combined with HA) outperformed bone marrow aspirate concentrate in cartilage regeneration quality [27]. Özdemir et al. (2023) and Çağlar Kir (2019) [23,24] reported that HA-based meshes enhance bone integration in orthopedic applications while Liu et al. (2023) [28] identified the activation of the BMP4/Smad1/5/8 signaling pathway as a key mechanism in HA-induced osteogenesis.

In the maxillofacial field, Göçmen et al. (2016) [29] concluded that HA increases bone height in maxillary sinus lift procedures, and Dogan et al. (2017) [30] showed that its combination with heterologous collagen significantly improves bone formation. Additionally, Kimball et al. (2024) [31] suggested that bone marrow stimulation combined with osteoconductive materials may optimize the repair of bone defects.

**Table 2:** Description of studies: population, interventions, and outcomes.

Author	Study Type	Number and Age of Patients	Methods	Results	Bone Regeneration	Bone Quality	Bone Density	Egree of Angiogenesis
Göçmen et al., 2016 [29]	Comparative split-mouth study	10 patients (6 women, 4 men), mean age: 56.7 years	Comparison of HA and ultrasonic resorbable pin fixation (URPF) for space maintenance in non-grafted sinus lifting. Measurement of height of alveolar bone (HAB), reduction in sinus volume (RSV), bone density, and implant survival.	Postoperative HAB and RSV were significantly greater on the URPF side. No statistically significant difference in implant survival or bone quality. A 100% implant survival rate. No complications observed.	Both techniques led to sufficient bone height for implant placement, but URPF resulted in higher HAB and RSV.	No type I bone identified; 35% in type II, 50% in type III, and 15% in type IV.	Comparable between both techniques.	Not specifically reported in the study.
Park et al., 2016 [25]	Open-label, single-arm, single-center, phase I/II clinical trial	7 patients, mean age: 58.7 years	Application of allogeneic umbilical cord blood-derived mesenchymal stem cells (hUCB-MSCs) combined with hyaluronic acid hydrogel for cartilage regeneration in osteoarthritic patients. Follow-up for 7 years, evaluating safety, VAS pain scores, IKDC subjective knee scores, MRI findings, and histological assessment.	Maturing repair tissue observed at 12 weeks; significant improvement in pain and knee function maintained over 7 years. No severe adverse effects reported. Histological evaluation showed hyaline-like cartilage. MRI at 3 years showed persistent cartilage regeneration.	Not directly assessed; focus was on cartilage regeneration.	Cartilage regeneration resulted in hyaline-like cartilage with good structural properties.	MRI findings indicated high glycosaminoglycan (GAG) content, suggesting strong cartilage quality.	Not specifically measured, but good integration with surrounding cartilage was reported.
Dogan et al., 2017 [30]	Randomized-controlled split-mouth study	13 patients (8 women, 5 men), age range: 33–69 years	Comparison of collagenated heterologous bone graft (CHBG) alone versus CHBG + hyaluronic matrix for sinus augmentation. Micro-CT and histomorphometric analysis performed on bone biopsy samples after 4 months.	Significantly higher percentage of new bone in CHBG + hyaluronic matrix group compared to CHBG alone. No implant loss observed.	CHBG + hyaluronic matrix enhanced bone formation compared to CHBG alone.	Higher osseous stiffness observed in CHBG + hyaluronic matrix group.	Higher bone density in CHBG + hyaluronic matrix group.	Increased vascularization observed in CHBG + hyaluronic matrix group.
Kir et al., 2019 [24]	Retrospective clinical study	Forty-four patients with atrophic midshaft clavicular nonunion, mean age 42.4 years	Comparison of iliac wing autograft with anatomical locking plate (ALP) fixation alone versus ALP + HA-based mesh. Assessed fracture healing time, clavicular length, Disabilities of the Arm, Shoulder and Hand (DASH) score, and constant score over a 2-year follow-up.	Group with HA-based mesh showed significantly shorter fracture healing time (14.7 vs. 19.6 months), higher constant score (90.2 vs. 81.5), and better DASH score (7.3 vs. 17.5) compared to the control group.	HA-based mesh improved bone healing by enhancing osteogenesis and maintaining periosteal integrity.	Better bone healing and structural integrity in the HA-based mesh group.	Faster mineralization and improved bone regeneration in the HA-based mesh group.	HA-based mesh stimulated angiogenesis, improving fracture healing.
Na et al., 2020 [27]	Retrospective comparative study	81 cases assessed, 31 cases with kissing lesion, 25 in BMAC group, 14 in Cartistem group	Comparison of the cartilage regeneration between bone marrow aspirate concentrate (BMAC) and allogeneic hUCB-MSCs (Cartistem) in medial unicompartmental osteoarthritis after high tibial osteotomy. Evaluated IKDC, KSS, WOMAC, and ICRS CRA grading system through secondary arthroscopy.	Both groups showed clinical improvement, but no significant differences in clinical and radiologic outcomes. However, Cartistem showed significantly better cartilage regeneration compared to BMAC in second-look arthroscopy (*p* = 0.002, 0.000).	Cartistem resulted in higher quality cartilage regeneration compared to BMAC.	Grade II cartilage observed in 85.7% of Cartistem group vs. 40% in BMAC group.	Higher cartilage integrity observed in Cartistem group based on ICRS CRA grading.	Not specifically reported in the study.
Velasco-Ortega et al., 2021 [26]	Randomized controlled trial	24 patients undergoing maxillary sinus augmentation, divided into three groups	Comparison of anorganic bovine bone mineral (ABBM), tricalcium phosphate (TCP), and TCP with hyaluronic acid (TCP+HA) for maxillary sinus augmentation. Evaluated histomorphometric, clinical, and patient-reported outcomes after 9 months.	No significant difference in percentage of new bone among groups. Residual biomaterial was significantly higher in the ABBM group, while TCP and TCP+HA groups had significantly lower nonmineralized tissue. Implant insertion torque was higher in the ABBM group.	All groups showed sufficient bone regeneration for implant placement.	Higher mineralized tissue observed in ABBM group, suggesting better mechanical resistance.	No significant difference among groups.	Increased vascularization observed around biomaterials in TCP and TCP+HA groups.
Liu et al., 2023 [28]	Experimental study (clinical and animal model)	20 patients with femoral fractures and rat femoral fracture model	Investigated the role of Hyaluronan and Proteoglycan Link Protein 1 (HAPLN1) in osteogenic differentiation and fracture healing. Used human serum samples, a rat femoral fracture model, and MC3T3-E1 osteoblast cell line. Examined BMP4/Smad1/5/8 signaling pathway involvement.	HAPLN1 was significantly overexpressed in fracture healing. Silencing HAPLN1 inhibited osteogenic differentiation and mineralization in MC3T3-E1 cells. BMP4/Smad1/5/8 pathway was identified as a key regulator in HAPLN1-induced osteogenesis.	HAPLN1 promoted osteoblast differentiation and fracture healing by activating BMP4/Smad1/5/8 signaling.	Enhanced bone formation and mineralization observed with increased HAPLN1 expression.	HAPLN1 knockdown reduced osteoblast mineralization and alkaline phosphatase activity.	Not specifically reported, but improved bone regeneration was observed.
Özdemir et al., 2023 [23]	Case report	Two cases: 42-year-old female with rheumatoid arthritis and glenoid defect; 19-year-old male with neglected Galeazzi fracture and radius non-union	Use of 3D-printed polycaprolactone (PCL) + hyaluronic acid-based scaffold for bone regeneration. One case involved reverse total shoulder arthroplasty, and the other involved open reduction and internal fixation of the radius.	Successful bone regeneration observed in both cases. The scaffold provided good integration, with no implant loosening or scaffold lysis over follow-up. Full range of motion achieved in both patients.	3D-printed scaffold facilitated bone regeneration and provided structural support.	Good bone integration with maintained mechanical stability.	Improved bone density in scaffold-treated areas.	Not specifically reported, but good bone healing was observed.
Kimball et al., 2024 [31]	Review study	Not specified, general review on bone marrow stimulation for osteochondral lesions	Analysis of bone marrow stimulation techniques for cartilage regeneration in osteochondral lesions of the talus. Discusses optimal lesion size, depth, and use of biological adjuvants.	Bone marrow stimulation is effective for lesions < 107.4 mm^2^ and <5 mm in depth. Larger lesions (>15 mm) require additional cartilage restoration techniques. Biological adjuvants improve outcomes.	Bone marrow stimulation induces cartilage repair but is limited by lesion size and depth.	Effectiveness depends on lesion chronicity and presence of cystic components.	Not directly measured but influenced by bone marrow stimulation success.	Biologic adjuvants can enhance angiogenesis and tissue repair.

### 3.3. Quality Assessment and Risk of Bias

The evaluated studies included different methodological designs, each assessed with specific tools for quality and risk of bias. For case series, evaluated using the JBI Critical Appraisal Tool, the included studies were those by Erdi Özdemir et al. (2023) [23], which reported the use of 3D scaffolds in orthopedics in two clinical cases, and Mustafa Çağlar Kir (2019) [24], which explored the effectiveness of a hyaluronic acid mesh in the treatment of atrophic nonunion of the clavicle (see Table 3). Regarding cohort and retrospective observational studies, assessed using the ROBINS-I tool, included works such as that of Seung-Min Na et al. (2020) [27], which compared the use of BMAC and Cartistem in patients with osteoarthritis, and Hu Liu et al. (2023) [28], focused on the analysis of HAPLN1 expression in osteogenic differentiation(see Table 4). Additionally, Jeff S. Kimball et al. (2024) investigated bone marrow stimulation in talus injuries [31], while Gökhan Göçmen et al. (2016) [29] assessed the comparison between the use of hyaluronic acid and resorbable pins in maxillary sinus lift procedures. E. Dogan et al. (2017) [30] conducted a comparison between heterologous collagen with and without hyaluronic acid in a similar context. Finally, the randomized clinical trials, evaluated with the ROB-2 tool, included the study by Yong-Beom Park et al. (2016) [25], which investigated cartilage regeneration through stem cells, and that of Eugenio Velasco-Ortega et al. (2021) [26], which compared various biomaterials used in maxillary sinus augmentation(see Table 5). This diversity of designs and topics provides a broad and detailed overview of the clinical applications of various treatments and techniques in orthopedics and bone regeneration.

### 3.4. GRADE Quality Assessment

This table includes the quality evaluation of all studies using the GRADE approach. It presents the study design, initial quality, risk of bias, inconsistency, indirectness, imprecision, publication bias, and overall quality rating for each of the studies assessed (see Table 6).

## 4. Discussion

Regenerating musculoskeletal tissues such as cartilage and bone remains a significant clinical challenge due to their limited intrinsic capacity for self-repair. In this context, HA has emerged as a multifunctional biomaterial with promising applications in bone and cartilage tissue engineering. Its biocompatibility, viscoelastic properties, and ability to modulate the cellular microenvironment make it particularly suitable as a carrier for mesenchymal stem cells (MSCs), morphogenetic proteins, and bone graft materials. Specifically, its combination with MSCs or bone grafts has demonstrated synergistic effects, enhancing both osteogenesis and chondrogenesis.

Several clinical studies have confirmed the potential of HA in real-world therapeutic scenarios. Park et al. (2017) [25] and Na et al. (2020) [27] reported that Cartistem, a formulation composed of HA hydrogel and human umbilical cord blood-derived MSCs (hUCB-MSCs)—significantly promoted the regeneration of articular cartilage in patients with Kellgren–Lawrence grade 3 osteoarthritis. These patients experienced sustained improvements in pain (measured by VAS) and joint function (IKDC) over a 7-year follow-up period [25], with Cartistem yielding superior cartilage quality compared to bone marrow aspirate concentrate (BMAC), as shown by the proportion of ICRS CRA grade II cartilage in the medial femur (85.7% vs. 40%) [27]. These findings suggest that allogeneic MSCs may offer advantages over autologous sources due to their greater homogeneity, proliferation capacity, and immediate availability.

In the field of bone regeneration, Velasco-Ortega et al. (2021) and Dogan et al. (2017) [26,30] found that HA combined with collagenated heterologous bone grafts (CHBGs) significantly enhanced new bone formation and mineralization in maxillary sinus lift procedures. Similarly, Özdemir et al. (2023) [23] demonstrated that HA incorporated into 3D-printed poly-ε-caprolactone (PCL) scaffolds produced favorable results in open surgical procedures such as osteotomies, although technical limitations still constrain its use in minimally invasive applications. Liu et al. (2023) [28] further demonstrated that HA combined with BMP-4 promoted osteogenesis via the activation of the Smad1/5/8 signaling pathway. Conversely, Göçmen et al. (2016) [29] reported that HA used in combination with bovine bone grafts did not offer additional regenerative benefits, emphasizing the need for the careful optimization of hydrogel composition.

The synergistic use of HA with MSCs or bone grafts appears to significantly enhance the osteoinductive and osteoconductive properties of the material. Park et al. (2016) [25] and Na et al. (2020) [27] reported increased bone density and the faster consolidation of critical-sized defects when HA was used in combination therapies. Additionally, Liu et al. (2023) [28] identified the HAPLN1 protein as a key regulatory element in osteogenesis, suggesting that certain extracellular matrix components associated with HA could serve as future therapeutic targets.

Despite these promising outcomes, significant challenges persist. While randomized controlled trials by Park et al. (2016) [25] and Velasco-Ortega et al. (2021) [26] support the safety and efficacy of HA—with a low incidence of adverse events—most of the supporting evidence comes from observational studies. These are inherently more prone to bias due to the absence of randomization and the presence of confounding variables [30,31]. Moreover, the lack of standardized HA formulations and wide variability in clinical application protocols complicate cross-study comparisons and limit the generalizability of the results.

### 4.1. Limitations

This systematic review has several notable limitations. First, there is substantial methodological and clinical heterogeneity among the included studies, particularly in study design, the formulations and concentrations of hyaluronic acid used, application protocols, and outcome measures. This heterogeneity precluded the possibility of conducting a quantitative meta-analysis, and a narrative synthesis was instead used to qualitatively describe and compare findings.

Although three randomized controlled trials were included (Park et al., 2016 [25]; Velasco-Ortega et al., 2021 [26]; Dogan et al., 2017 [30]) the majority of the evidence is derived from observational or lower-quality studies, which are more susceptible to bias and reduce the overall strength and reliability of the conclusions. Another important limitation is the lack of a standardized HA formulation—regarding molecular weight, cross-linking degree, purity, or concentration—which makes it difficult to establish clear and reproducible clinical guidelines. Future studies should systematically report the range of HA concentrations used, as this may significantly influence clinical outcomes.

Furthermore, some of the included studies primarily focused on cartilage regeneration rather than bone healing, which may reduce the relevance of the findings for orthopedic and dental applications. Lastly, there is a marked absence of RCTs specifically investigating the use of HA in minimally invasive foot and ankle surgery. This represents a critical gap in the literature and underscores the need for high-quality, well-designed clinical trials that address this specific surgical context.

### 4.2. Implications for Practice, Policy, and Future Research

The findings of this review suggest that HA, especially when combined with biomaterials or stem cells, holds significant potential for enhancing bone regeneration and fracture healing. Clinically, this could lead to the development of new HA-based treatments tailored for use in minimally invasive surgical procedures, reducing recovery times and improving patient outcomes. From a policy perspective, the integration of HA into standard bone healing protocols may warrant evaluation through cost-effectiveness analyses and clinical guidelines.

However, a notable gap remains in the literature regarding the specific application of HA in minimally invasive foot and ankle surgeries. To date, no high-quality randomized controlled trials (RCTs) have directly assessed the role of HA in enhancing bone consolidation following MIS foot osteotomies. This underscores the need for targeted research in this area. We propose the design of a multicenter, randomized controlled trial comparing standard postoperative care with and without the adjunctive use of HA in patients undergoing MIS distal metatarsal osteotomies. Primary outcomes should include time to radiographic consolidation, postoperative pain (e.g., via VAS), and functional recovery (e.g., AOFAS score) at 6 and 12 weeks postoperatively. Such a study could provide high-level evidence to inform clinical guidelines and optimize patient care in foot and ankle surgery.

## 5. Conclusions

In conclusion, although HA demonstrates strong potential in bone regeneration, continued research is necessary to establish standardized protocols and determine the most effective combinations of biomolecules and delivery formats. In particular, its potential applications in podiatric surgery, especially for minimally invasive techniques, warrant further investigation, given the growing interest in biologically enhanced interventions within this field. Future controlled clinical trials with consistent study designs will be essential to validate these preliminary findings and to define the precise clinical indications for the use of HA in modern podiatric and orthopedic surgical practice.

## Figures and Tables

**Table 1:** Detailed summary of the systematic review methodology.

Section	Methodology
Search Strategy	Databases: PubMed, EbscoHost, Web of Science, and Scopus. Search string: (“Hyaluronic Acid” OR Hyaluronan OR “Hyaluronate alginate” OR “Sodium Hyaluronate” OR “Hyaluronic Gel” OR Hyaluronate OR Hyaluronidase) AND (“bone regene*” OR “Fracture Healing” OR Osteogenesis OR “Bone Formation” OR “Bone Repair” OR “Bone Healing” OR “Bone Regeneration”) AND (Osteotom* OR “Orthopedic Procedures” OR “Percutaneous Procedures” OR Osteosynthes* OR “Bone Surgery” OR “Fracture Fixation” OR “Bone Fracture Treatment” OR Orthopedics). Boolean operators and controlled vocabulary were used.
Search Period	Time frame: Last 10 years. Final search dates: 25–28 February 2025.
Study Selection	Two authors (H.F.S. and E.C.O.P.) independently reviewed titles and abstracts. The SR-Accelerator tool was used to resolve disagreements, with a third reviewer (J.F.T.) consulted when necessary. Mendeley was used to remove duplicate references.
Tools Used	Online deduplication tool: SR-Accelerator Deduplicator. Excel for data management. RoB 2, ROBINS-I, JBI, and GRADE tools to assess methodological quality and risk of bias.
Inclusion Criteria	Human studies published in the last 10 years. Focus on HA applied to bone consolidation in fractures and osteotomies. Use of imaging techniques and histological analysis to evaluate bone regeneration.
Exclusion Criteria	Studies lacking sufficient information on HA application. Animal studies without clinical correlation in humans. Systematic reviews or meta-analyses without primary data. Publications in languages other than English or Spanish. Studies in which HA was not the primary therapeutic component under evaluation.
Data Extraction	Two authors independently extracted and coded the following information: Author and year of publication. Type of HA used. Study design and applied interventions. Outcomes related to bone regeneration, bone quality and density, and angiogenesis.
Synthesis Approach	Narrative synthesis combined with quantitative analysis. Effect sizes, certainty of evidence, and statistical outcomes were analyzed. Heterogeneity in study design and interventions was taken into account.
PICO Assessment	Population: Adult patients with fractures or osteotomies. Intervention: Application of HA alone or in combination with bone grafts or other biomolecules. Comparison: Standard bone regeneration treatments without HA. Outcomes: Evaluation of accelerated bone consolidation, improved bone quality, graft integration, and angiogenesis.

**Table 3:** Quality and risk of bias assessment in case series (JBI Critical Appraisal).

Author/Year	it. 1	it. 2	it. 3	it. 4	it. 5	it. 6	it. 7	it. 8	it. 9	it. 10	Overall Quality
Özdemir et al. (2023) [23]	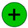	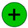	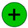	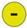	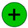	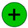	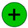	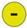	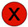	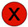	High risk
Kimball et al. (2024) [31]	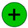	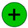	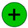	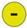	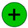	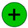	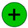	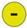	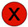	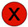	High risk
Kir et al. 2019 [24]	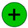	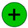	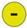	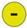	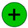	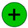	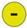	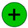	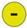	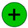	Moderate

The assessment of case series studies using the JBI Critical Appraisal tool is based on the following criteria: it. 1: Were clear inclusion criteria established for the case series? it. 2: Was the condition measured consistently and reliably for all participants included in the study? it. 3: Were valid methods used to identify the condition in all participants of the case series? it. 4: Did the study ensure the consecutive inclusion of participants? it. 5: Were all eligible participants fully included in the case series? it. 6: Was there a clear and detailed report of the demographics of the participants involved in the study? it. 7: Was clinical information about the participants reported transparently? it. 8: Were the outcomes or follow-up results of the cases reported clearly? it. 9: Was there transparent reporting of the demographic information of the presenting sites or clinics? it. 10: Was the statistical analysis conducted appropriately? In the visual representation of the assessment, green color (yes) indicates that the criterion was fully met; yellow color (unclear) suggests some ambiguity or insufficient information; red color (no) highlights a significant issue or that the criterion was not met.

**Table 4:** Risk of bias assessment in non-randomized studies (ROBINS-I).

Author/Year	it. 1	it. 2	it. 3	it. 4	it. 5	it. 6	it. 7	it. 8
Göçmen et al., 2016 [29]	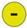	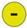	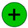	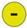	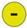	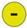	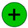	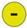
Na et al., 2020 [27]	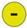	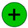	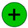	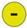	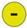	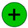	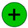	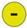
Liu et al., 2023 [28]	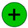	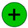	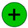	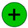	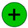	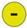	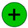	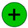

The evaluation of bias in non-randomized studies using the RoB 1 tool involves assessing the following criteria: it. 1: Was there potential bias due to uncontrolled confounding variables that could affect the study’s validity? it. 2: Was there bias in the selection of participants, indicating that the compared groups may not have been appropriately matched or representative of the target population? it. 3: Was there bias in the classification of interventions, ensuring that exposures or treatments were assigned correctly and consistently? it. 4: Was there bias due to deviations from intended interventions, where participants may not have adhered strictly to the assigned treatment protocols? it. 5: Was there bias due to missing data, suggesting that data loss or incomplete data could potentially influence the study’s results? it. 6: Was there bias in the measurement of outcomes, where the methods used for assessing outcomes might have introduced systematic errors? it. 7: Was there bias in the selection of reported results, indicating that only specific outcomes were selectively reported, potentially distorting the study’s findings? it. 8: Was the overall risk of bias evaluated by considering all domains collectively to provide a comprehensive assessment of the study’s validity?

**Table 5:** Risk of bias assessment in randomized controlled trials (ROB-2).

Author/Year	it. 1	it. 2	it. 3	it. 4	it. 5	it. 6
Park et al., 2016 [25]	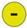	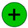	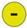	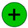	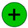	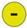
Dogan et al., 2017 [30]	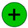	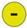	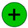	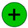	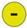	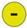
Velasco-Ortega et al., 2021 [26]	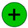	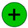	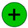	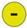	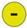	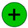

The ROB-2 tool is used to assess the risk of bias in randomized clinical trials, focusing on the following key domains: it. 1: Bias due to randomization: Was the randomization process implemented correctly to ensure that the groups being compared were comparable at baseline? Proper randomization helps minimize selection bias and confounding factors. it. 2: Bias due to deviations from intended interventions: Were there any deviations from the assigned interventions that could potentially impact the validity of the study? This assesses whether participants adhered to the treatment protocols as intended. it. 3: Bias due to missing data: Was the handling of missing data appropriate, or could the absence of data introduce bias into the study’s results? The proper management of missing data is critical to maintaining the integrity of the findings. it. 4: Bias in measurement of outcomes: Were outcome assessments conducted in a way that minimized measurement bias? This involves evaluating whether the methods used to measure outcomes were objective and consistent across all participants. it. 5: Bias in selection of reported results: Was there selective reporting of results, potentially leading to a misrepresentation of the study’s findings? This domain focuses on whether the researchers reported all relevant outcomes or only those that supported their hypotheses. it. 6: Overall risk of bias: This is the final assessment that considers the cumulative effect of all bias domains, providing an overall evaluation of the study’s methodological quality. Interpretation of risk levels: “Low” risk of bias (green): Indicates minimal concerns regarding the study’s validity, suggesting that the study’s findings are likely to be reliable. “Moderate” risk of bias (yellow): Reflects some concerns that could potentially impact the reliability of the results, highlighting areas where the study methodology could have been stronger. “High” risk of bias (red): Suggests serious methodological concerns that could significantly affect the study’s conclusions, indicating a need for caution when interpreting the results.

**Table 6:** GRADE quality assessment.

Author/Year	Study Design	Initial Quality	Risk of Bias	Inconsistency	Indirectness	Imprecision	Publication Bias	Final Quality
Göçmen et al., 2016 [29]	Observational	Low	Moderate	Moderate	High	Low	High	Low
Park et al., 2016 [25]	RCT	High	Moderate	Moderate	High	High	High	Moderate
Dogan et al., 2017 [30]	RCT	High	Moderate	Low	High	Low	High	Moderate
Kir et al., 2019 [24]	Observational	Low	High	High	Low	Moderate	Moderate	Low
Na et al., 2020 [27]	Observational	Low	Moderate	Moderate	Moderate	Moderate	Moderate	Low
Velasco-Ortega et al., 2021 [26]	RCT	High	High	High	Moderate	High	High	High
Liu et al., 2023 [28]	Observational	Low	High	High	Moderate	High	Low	Low
Özdemir et al., 2023 [23]	Quantitative	Moderate	Moderate	Low	Low	High	High	Moderate
Kimball et al., 2024 [31]	Quantitative	Moderate	Moderate	Moderate	Low	Moderate	High	Moderate

## Data Availability

Data are contained within this article.

## Abbreviations

The following abbreviations are used in this manuscript:

Abbreviation	Meaning
ABBM	Anorganic Bovine Bone Mineral
BMAC	Bone Marrow Aspirate Concentrate
BMP	Bone Morphogenetic Protein
CHBG	Collagenated Heterologous Bone Grafts
GAG	Glycosaminoglycan
GRADE	Grading of Recommendations Assessment, Development, and Evaluation
HA	Hyaluronic Acid
HAPLN1	Hyaluronan and Proteoglycan Link Protein 1
hUCB-MSCs	Human Umbilical Cord Blood-Derived Mesenchymal Stem Cells
ICRS CRA	International Cartilage Repair Society Cartilage Repair Assessment
IKDC	International Knee Documentation Committee
JBI	Joanna Briggs Institute
micro-CT	Micro-computed Tomography
MIS	Minimally Invasive Surgery
MSCs	Mesenchymal Stem Cells
OA	Osteoarthritis
PCL	Poly-ε-caprolactone
PICO	Population, Intervention, Comparison, Outcome
PRISMA	Preferred Reporting Items for Systematic Reviews and Meta-Analyses
RCT	Randomized Controlled Trial
ROB-2	Risk of Bias Tool for Randomized Trials
ROBINS-I	Risk of Bias In Non-randomized Studies-of Interventions
TCP	Tricalcium Phosphate
URPF	Ultrasonic Resorbable Pin Fixation
VAS	Visual Analog Scale

## Appendix A

Searches strings for electronic databases.
The original search string was created in PubMed. The searches strings employed for Web of Science and MEDLINE via PubMed were translated using an automatic online tool (https://sr-accelerator.com/#/polyglot). Filters: 10 years and human.
1. Search strategy for PubMed. URL: https://pubmed.ncbi.nlm.nih.gov on 25–27 February 2025
(“Hyaluronic Acid” OR Hyaluronan OR “Hyaluronate alginate” OR “Sodium Hyaluronate” OR “Hyaluronic Gel” OR Hyaluronate OR Hyaluronidase) AND (“bone regene*” OR “Fracture Healing” OR Osteogenesis OR “Bone Formation” OR “Bone Repair” OR “Bone Healing” OR “Bone Regeneration”) AND (Osteotom* OR “Orthopedic Procedures” OR “Percutaneous Procedures” OR Osteosynthes* OR “Bone Surgery” OR “Fracture Fixation” OR “Bone Fracture Treatment” OR Orthopedics)
Search strategy for Web of Science URL: https://www.webofscience.com/wos/alldb/basic-search on 25–27 February 2025
(“Hyaluronic Acid” OR Hyaluronan OR “Hyaluronate alginate”) AND (“bone regene*” OR “Fracture Healing” OR Osteogenesis) AND (Osteotom* OR “Orthopedic Procedures” OR “percutaneous”)
Search strategy for Scopus
(TITLE-ABS-KEY (“Hyaluronic Acid” OR hyaluronan OR “Hyaluronate alginate”) AND TITLE-ABS-KEY (“bone regene*” OR “Fracture Healing” OR osteogenesis) AND TITLE-ABS-KEY (osteotom* OR “Orthopedic Procedures” OR “percutaneous”))
Search EBSCOH
(‘hyaluronic acid’ OR hyaluronan OR ‘hyaluronate alginate’) AND (‘bone regene*’ OR ‘fracture healing’ OR osteogenesis) AND (osteotom* OR ‘orthopedic procedures’ OR percutaneous)
